# One-year clinical results of restorations using a novel self-adhesive resin-based bulk-fill restorative

**DOI:** 10.1038/s41598-022-07965-z

**Published:** 2022-03-10

**Authors:** Andreas Rathke, Frank Pfefferkorn, Michael K. McGuire, Rick H. Heard, Rainer Seemann

**Affiliations:** 1 Dentsply Sirona, Konstanz, Germany; 2 University of Ulm, Faculty of Dentistry, Ulm, Germany; 3 The McGuire Institute, Houston, TX USA; 4 Department of Restorative, Preventive and Pediatric Dentistry, zmk Bern, University of Bern, Bern, Switzerland

**Keywords:** Dentistry, Clinical trial design

## Abstract

This prospective study assessed the dual-curing self-adhesive bulk-fill restorative Surefil one. The restorations were placed and reviewed by dental practitioners who are members of a practice-based research network in the United States. Seven practitioners filled 60 cavities (20 class I, 19 class II and 21 class V) in 41 patients with Surefil one without adhesive, according to the manufacturer’s instructions. The restorations were evaluated using modified USPHS criteria at baseline, 3 months, and 1 year. Patients were also contacted to report postoperative hypersensitivity one to four weeks after placement. The only patient that showed moderate hypersensitivity after 1 year had previously reported symptoms that were unlikely associated to the class I molar restoration. One class II restoration in a fractured maxillary molar was partially lost. The remaining restorations were found to be in clinically acceptable condition resulting in an annual failure rate of 2%. Color match showed the lowest number of acceptable scores (88%) revealing significant changes over time (P = 0.0002). No significant differences were found for the other criteria (P > 0.05). The novel self-adhesive bulk-fill restorative showed clinically acceptable results in stress-bearing class I and II as well as non-retentive class V cavities at 1-year recall.

## Introduction

Resin-based composites have become the standard filling material in dental practices for anterior and posterior restorations. Long-term clinical studies confirmed that the longevity of direct composite restorations in posterior teeth is comparable to that of amalgam restorations^1–4^. In addition, innovations in composite technology have simplified the application. Compared to conventional composite application in 2 mm thick layers, bulk-fill composites can be placed in 4–5 mm layer thickness due to their reduced polymerization shrinkage stress and high reactivity to light curing^5,6^. Clinical data of up to 10 years confirmed the safe applicability of these bulk-fill composites as alternative to conventional posterior composite restorations^6–8^. Further simplification involved the development of self-adhesive composites that eliminated the use of an adhesive, thus minimizing the time in which blood or saliva contamination could compromise the restoration. The most common approach was modifying the reactive diluents with acidic moieties to facilitate the bonding with enamel and dentin. This approach was commercialized as self-adhesive flowable composites, but many laboratory studies have questioned whether these materials are a valid alternative to composites where a separate adhesive is applied^9–11^. Particularly in load-bearing areas, the contradictory clinical performance of self-adhesive restorative materials has not led to a breakthrough^12–16^.

Alternatively, the structural monomers can be modified with acidic groups to achieve sufficient adhesion. To its extreme this approach is realized in the polyacids used in glass ionomer cements^17^. However, polyacids cannot contribute to the radically polymerized network due to lack of polymerizable groups. Recently, a modified polyacid system of high molecular weight (MOPOS) has been formulated and patented to merge the self-adhesive properties of classical polyacids known from glass ionomer cements with the crosslinking ability of structural monomers known from composites^18^. The self-adhesive resin-based bulk-fill restorative (classified as self-adhesive composite hybrid by the manufacturer) has been launched under the brand name Surefil one (Dentsply Sirona, Konstanz, Germany). The manufacturer describes the initiator system as a combination of the photoinitiator camphorquinone and a persulfate with two reducing agents both being part of the dark as well as the light curing process. This leads to bulk curing (in the dark) as well as light curing of the surface areas^18^.

In vitro research confirmed comparable mechanical properties (flexural strength, fatigue strength, flexural modus, and fracture toughness) of the self-adhesive bulk-fill restorative to clinically established posterior restorative materials and similar or better wear resistance to newer self-adhesive restoratives^19–21^. The level of self-adhesiveness to enamel and dentin was comparable to contemporary adhesives and glass ionomer cements^22,23^. However, limited information is available on the clinical performance of the novel self-adhesive bulk-fill restorative. Thus, the aim of this practice-based research network (PBRN) study was the prospective follow-up of the self-adhesive bulk-fill restorations placed in daily practice under 'real world' conditions. The null hypothesis to be tested was that there are no changes in performance over the 1-year observation time.

## Methods

### Study design and population

The direct restorations were placed and reviewed by seven experienced general dental practitioners (GDPs) who were members of a PBRN in the United States (The McGuire Institute, Houston, TX). The GDPs practiced in Houston and Missouri City (TX, USA). The Advarra Institutional Review Board in Columbia (Maryland, USA) approved the study (Protocol Number 00036511). All methods were conducted in accordance with good clinical practice, national guidelines, and regulations. A total of 41 patients from the dental practices (21 female, 20 male) satisfying the inclusion and exclusion criteria were enrolled. The patients were included into the study if they were over 18 years of age, required at least one class I, II or V direct restoration in permanent teeth having a positive sensitivity test, had dentition free of active periodontal disease and rampant caries, and were in good general health. The patients were excluded from the study if they had language barriers, severe medical conditions or drug use, allergic history concerning methacrylate, lack of compliance, or were pregnant. The average age of patients was 55.4 years with a range of 21–78 years. All patients participated voluntarily and were required to provide informed written consent with having the right to withdraw from the clinical study at any time. Each practice placed ten restorations. One practice with two GDPs performed five restorations each. Reasons for placement were replacement restorations (n = 24) and caries lesions (n = 36). All restorations were inserted in teeth that did not require direct pulp capping and showed no hypersensitivity preoperatively, except one tooth with slight sensitivity (defined as occasional but not uncomfortable). Twenty-two patients received one restoration and 19 patients two restorations. The sample size was based on previous similar study designs and recommendations of at least 50 restorations per material with maximum two restorations per patient at baseline^24^. The distribution of the involved teeth and restorations is detailed in Table 1.

**Table 1:** Distribution of the self-adhesive bulk-fill restorations at baseline (n = 60).

Total	Maxillary			Mandibular	
	Canine	Premolar	Molar	Premolar	Molar
**Class I**^**a**^					
20	1	2	14	2	1
**Class II**					
19	0	7	5	5	2
**Class V**					
21	2	5	0	10	4
**Total**					
60	3	14	19	17	7

^a^Occlusal surface of premolars and molars, lingual surface of canine.

### Restorative procedure

After a web-based introduction to the self-adhesive bulk-fill restorative, the GDPs applied the material on model teeth to become familiar with its handling properties. The composition of the restorative material is given in Table 2.

**Table 2:** Composition of the self-adhesive bulk-fill restorative as per manufacturer.

Material (manufacturer)	Composition	Lot number	Application
Surefil one (Dentsply Sirona, Konstanz, Germany)	Aluminium-phosphor-strontium-sodium-fluoro-silicate glass, water, highly dispersed silicon dioxide, acrylic acid, polycarboxylic acid (MOPOS), ytterbium fluoride, bifunctional acrylate (BADEP), self-cure initiator, iron oxide pigments, barium sulfate pigment, manganese pigment, camphorquinone, stabilizer	Shade A3: 1807004175	Bulk application, dual-curing

At the time of restoration placement, only the shade A3 was available. Where indicated, local anesthesia was administered. The outline of the cavity was determined by the size of the restoration to be replaced and/or the lesion after caries removal (defect-oriented preparation). The cavity was then finished using fine diamonds or carbides to reduce the smear layer. No undercuts or bevels were prepared. The size of the cavity was assessed with dental explorers. The isthmus width of class I and II cavities was ≤ 1/3 (n = 25) and ≤ 2/3 (n = 14) of the intercuspal distance, respectively. Dentin thickness between cavity floor and pulp was estimated from radiographs. The distance was less than 1 mm in seven cavities, between 1 and 2 mm in 21 cavities, and more than 2 mm in 32 cavities. In two cavities, a hard-setting calcium hydroxide liner (Dycal, Dentsply Sirona) was used to selectively cover the dentin close to the pulp. Isolation of the operative field was achieved either with cotton rolls or, in three cases, with rubber dam. Matrices and wedges were selected depending on the cavity class and personal preferences of the GDPs placing the restoration. The cavities were cleaned by air–water spray leaving a moist cavity surface. The activated capsules (Surefil one) were mixed for 10 s using a capsule mixer according to the manufacturer’s instructions. The self-adhesive material was dispensed immediately into the cavity from the capsule tip using a capsule extruder, starting dispensing at the deepest portion of the cavity, and keeping the tip close to the cavity floor. The tip was gradually withdrawn as the cavity was filled in bulk and contoured with a hand instrument. Number of capsules applied depended on the cavity size. After the material was set (approximately in 6 min) or optionally light cured on the restoration surface for 20 s, the occlusal contacts were evaluated with marking paper. The light-curing units were used from the practices’ inventory and had to meet the minimum requirement by the manufacturer's instructions for use (radiant emittance ≥ 800 mW/cm^2^ and camphorquinone absorption spectrum with a peak between 440 and 480 nm). The finishing and polishing with silicon instruments (Enhance Finishing System, Dentsply Sirona) was performed in the same session keeping the restoration moist using air–water spray.

### Evaluation procedure

The restorations were placed between January and March 2019, and examined at baseline, 3 months, and 1 year. Patient’s 1-year recall was performed January up to August 2020 (mean service time 394 ± 44 days) due to the COVID-19 pandemic. Registration and case report forms were completed after placement of the restorations and at follow-up visits. To evaluate the immediate postoperative hypersensitivity, patients were contacted by telephone, text, or e-mail once a week after the placement for four weeks. These interviews were used as follow-up procedure to minimize recall loss as the patient was not required to return to the practice until the 3-month recall. However, patients were instructed to visit the practice if they had any discomfort. At the recalls, the patients were asked again about persisting or new hypersensitivity. The USPHS criteria were selected^24^ and modified to reflect the practitioners' way of rating their restorations (Table 3). Evaluation tools were dental mirrors, explorers, magnifying glasses, and intraoral photographs. Radiographs were only taken if clinically indicated, for example caries diagnostic or pain interpretation. Adverse events regarding product safety were recorded.

**Table 3:** Modified USPHS criteria for evaluation of the self-adhesive bulk-fill restorations.

Criteria	
Restoration quality	0 = Intact
	1 = Chipping
	2^a^ = Fracture
	3^a^ = Loss
Marginal quality	0 = Smooth
	1 = Step
	2^a^ = Gap
Tooth quality	0 = Sound
	1 = Cracking
	2^a^ = Fracture
Proximal contact	0 = Yes (Class II only)
	1^a^ = No (Class II only)
Caries	0 = No
	1^a^ = Yes
Vitality	0 = Yes
	1^a^ = No
Hypersensitivity	0 = No sensitivity is experienced at any time
	1 = Slight sensitivity is experienced occasionally but it is not uncomfortable
	2^a^ = Moderate sensitivity is experienced intermittently, and it is uncomfortable
	3^a^ = Severe discomfort is noted routinely with cold or pressure stimulation
Color match	0 = Perfect color match
	1 = Good color match
	2 = Slight color mismatch
	3^a^ = Obvious color mismatch
	4^a^ = Not at all satisfied
Color match (patient view)	0 = Perfect color match
	1 = Good color match
	2 = Slight color mismatch
	3^a^ = Obvious color mismatch
	4^a^ = Not at all satisfied

^a^Unacceptable scores.

### Statistical analysis

Statistical unit was one restoration. The endpoint of the restoration, i.e., the need for replacement or repair, was defined as clinical failure. Data were analyzed with the Statgraphics Centurion XVI 16.2.04 (Statgraphics Technologies, Inc., The Plains, Virginia, USA). The null hypothesis was tested by the non-parametric Wilcoxon signed-rank test for continuous variables and ordered categorical data and by means of the Binomial test for binary data. Two-sided P-values below 5% were considered indicating that the results were statistically significant.

### Ethical approval

All procedures performed in studies involving human participants were in accordance with the ethical standards of the institutional research committee and with the 1964 Helsinki declaration and its later amendments or comparable ethical standards.

### Informed consent

Written informed consent was obtained from all individual participants included in the study.

## Results

**Figure 1:**
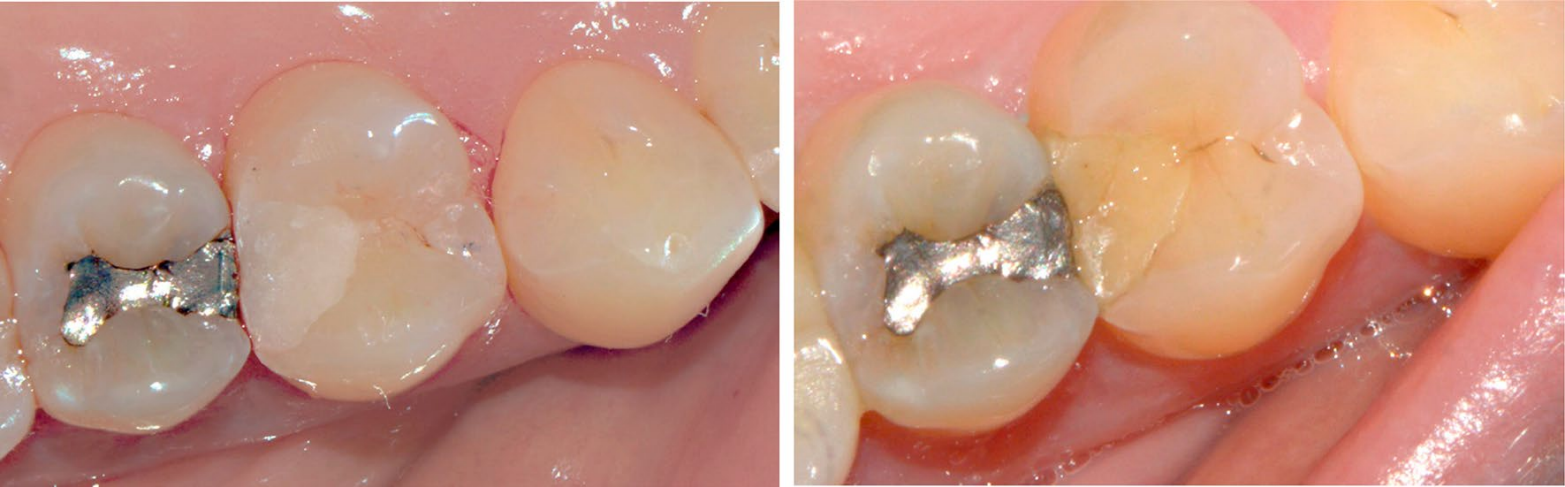
Class II restoration in upper first premolar at baseline (left) and 1-year recall (right). Color change and occlusal steps at the enamel margin were visible after 1 year.

**Figure 2:**
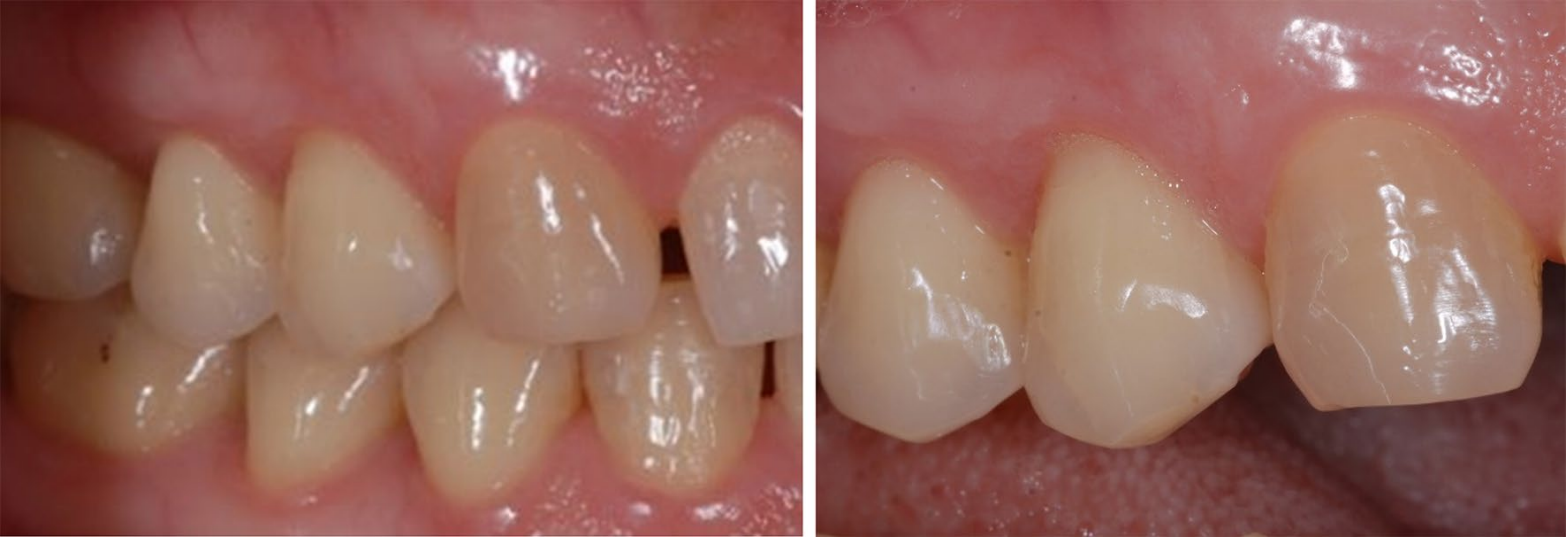
Class V restoration on upper first premolar at baseline (left) and 1-year recall (right) without any visible change.

**Figure 3:**
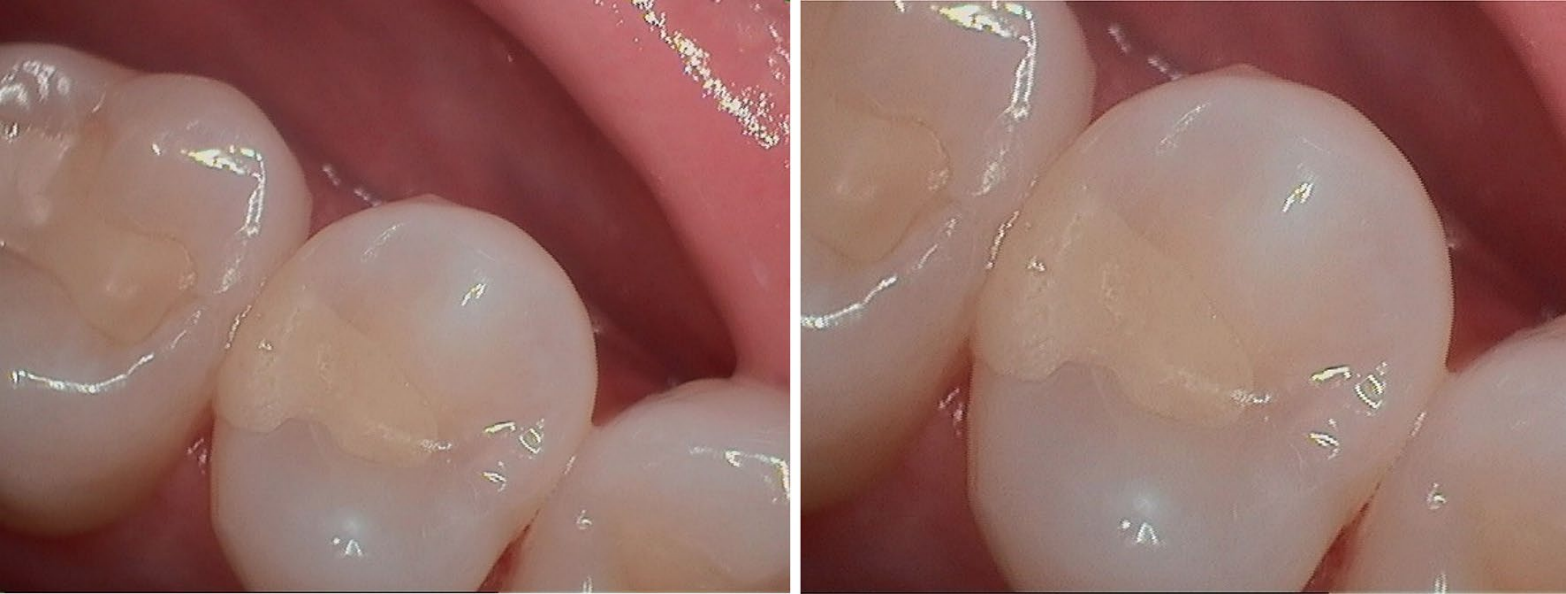
Class II restoration in second upper premolar at 1-year recall (left) with no visible sign of surface roughness (right).

Dropouts occurred for one patient with one class V restoration after 3 months and for seven patients with 11 restorations (four class II and seven class V) after 1 year, which results in recall rates of 98% and 82%, respectively. After 1 year, 41 (84%) restorations did not show any of the unacceptable scores listed in Table 3. The only patient that showed moderate hypersensitivity after 1 year (2%) had also reported symptoms at the 3-month recall that were unlikely associated to the class I molar restoration. One lower premolar was reported as non-vital and fractured distally to the buccal class V restoration which remained intact and was not considered as reason for failure. One class II restoration in a fractured maxillary molar was partially lost resulting in an annual failure rate of 2%. Illustrations of a representative sample of restorations are presented in Figs. 1, 2 and 3. No adverse events associated with the use of the restorative material (other than the failure rate) were observed. The lowest number of acceptable scores after 1 year was found for color match (88%). However, the color match of restorations significantly improved over time (P = 0.0002). No significant differences were found for the other evaluated criteria (P > 0.05) (Table 4).

**Table 4:** Results (%) of the self-adhesive bulk-fill restorations evaluated at baseline and the follow-up visits.

Criteria	Baseline (n = 60)	Three-month recall (n = 59)	One-year recall (n = 49)	Failure
**Restoration quality**				
0	100	100	94	
1	0	0	4	
2	0	0	0	
3	0	0	2	n = 1
**Marginal quality**				
0	97	98	88	
1	0	0	8	
2	3	2	4	
**Tooth quality**				
0	98	100	98	
1	2	0	0	
2	0	0	2	
**Proximal contact**				
0	95	100	100	
1	5	0	0	
**Caries**				
0	100	100	100	
1	0	0	0	
**Vitality**				
0	100	100	98	
1	0	0	2	
**Hypersensitivity**				
0	100	98	96	
1	0	0	2	
2	0	0	2	
3	0	2	0	
**Color match**				
0	5	17	25	
1	10	37	16	
2	42	22	47	
3	28	17	12	
4	15	7	0	
**Color match (patient view)**				
0	–	42	51	
1	–	37	18	
2	–	14	23	
3	–	7	8	
4	–	0	0	

Significant changes over time were found for color match (P = 0.0002)—not determined.

## Discussion

This prospective practice-based research network (PBRN) study evaluated the dual-curing self-adhesive resin-based bulk-fill restorative Surefil one, which is indicated as a permanent filling material for class I to V restorations. The novel restorative underwent thorough preclinical screening with encouraging results in terms of bond durability, long-term mechanical stability, and wear resistance. The individual studies were previously published in a compilation on ‘Self-adhesive restorative materials—State-of-the-Art’^19–23,25^.

A practice-based setting was chosen to investigate the material's clinical performance during daily routine treatment, i.e., its effectiveness^26^. The survival rate of posterior composite restorations tended to be lower in dental practice under 'real world' conditions than predicted in university-based studies under ideal conditions for composite placement^27^. The seven participating general dental practitioners (GDPs) in this study were part of a United States-wide PBRN consisting of 23 private practice surgical investigators at 17 sites with 980 referrers and 15 restorative sub-investigators at 12 sites representing a wide variety of dentists and having conducted over 24 clinical studies to date. During the material's training, all seven GDPs were briefed on the case report form and the evaluated criteria. The evaluation of the restorations was done by the GDP who placed the restoration and not by independent and calibrated dentists. This may have caused difficulties to control evaluation bias and confounders. In daily practice, however, the decision for clinical intervention (refine, repair, or replace) is not made by a third party. The method of evaluating restorations by the treating dentist instead of a blinded and calibrated third party has also been used in some other PBRN studies^28,29^. Although the evaluated criteria were not as strict as in university-based studies and were lacking a comparison group, the fact the material was used by GDPs may have given the results a realistic validity^26^. A clinical evaluation of surface roughness or wear was not performed since visual inspection can only detect gross differences in wear loss. No such evidence was found in the wear studies^19,20^ or in this study so far (Fig. 3).

The self-adhesive material was applied in bulk into the cavities without beveling the margins or applying additional adhesive and etching steps, as specified by the manufacturer's instructions for use. Selective etching of enamel margins with phosphoric acid was not performed, because this would have required an additional working step on enamel and could interfere with the self-adhesion on dentin, at least in the case of self-adhesive resin luting cements^30^. However, compared to selective enamel etching, self-etch/self-adhesive formulations showed lower enamel bond strength, particularly on uncut enamel^30,31^. Enamel beveling was therefore not prepared as beveled margins are difficult to detect and resin flash or overhangs on uncut enamel would have been more likely to result in marginal staining and chipping during clinical service. Cotton rolls isolation during restoration placement was used in most of the cases (95%), which reflected daily practice and complied with the manufacturer's instructions for use.

When the follow-up periods were analyzed, postoperative hypersensitivity within an unacceptable range (scores 2 and 3) was 0% at baseline, 4% at two weeks, and 2% at the following recall timepoints. Postoperative hypersensitivities of 4–14% were reported in the first week after composite placement, decreasing over the weeks and with an occurrence being higher in deeper cavities and Class II restorations^32^. Postoperative hypersensitivity of a self-adhesive flowable composite was comparable with that of a hybrid composite in conjunction with a self-etching adhesive, decreasing two weeks after class I restoration placement^33^. In another clinical study, postoperative hypersensitivities of self-adhesive bulk-fill restoratives decreased over 1 month from 4.2 to 0% for Activa Bioactive-Restorative, from 12.5 to 4.2% for Equia Forte, and from 29.2 to 10.4% for Cention N, respectively^34^. The present study confirms previous evaluations, in which a Germany-based group of 24 dentists placed over 1200 Surefil one restorations mostly in class I and II cavities of over 1000 patients and reported 0.8% hypersensitivity postoperatively^35^. The low values of postoperative hypersensitivity could be attributed to sufficient self-adhesive properties of the material with no additional etching or adhesive step in deeper dentin parts near to the pulp as well as reduced polymerization shrinkage stress and adequate curing depth even in deeper layers of the restoration. According to the manufacturer the key component of Surefil one is the hydrolytically stable MOPOS monomer, which both promotes adhesion to enamel and dentin and acts as a copolymerizing crosslinker in the cured material^18,35^. The bonding mechanism is primarily based on chemical (ionic) bond between the carboxylic acid groups in both MOPOS and acrylic acid to calcium ions of the hydroxyapatite. A micromechanical bond through infiltration of the smear layer and surface demineralization or hybridization could also contribute to self-adhesion. According to the manufacturer, the pH is 2.1 directly after mixing and 3.2 after 6 min. The material then gradually becomes neutral. Adhesive interface micromorphology demonstrated a close interaction with smear-covered dentin (wet ground by 800-grit sandpaper, roughness corresponding to the finishing with a fine diamond bur) and small resin tags within dentin tubules, whereas Activa Bioactiva-Restorative and Cention N showed interfacial gaps and significantly lower self-adhesion to dentin. However, dentin bond strengths were higher for the self-adhesive materials when used with a universal adhesive in self-etch mode^36^. It is worth mentioning that over-wet surfaces would dilute acids, leading to decreased infiltration of the smear layer, while overdried dentin can be compensated for by the water in the powder-liquid formulation^23^.

The clinical performance of the restorations having only a slight decrease in marginal quality from 97% acceptable (scores 0 and 1) to 96% within the first year of clinical service supported in vitro data on bond strength and marginal quality of Surefil one restorations after aging procedures^19,22,25^. In particular, this applied for the 100% retention rate of the 14 class V restorations, as their lesions were non-retentive indicating the material's self-adhesive effectiveness. Çelik et al. evaluated a self-adhesive flowable composite in non-carious cervical lesions for 6 months. A 33% retention rate was observed when compared with 100% for a hybrid composite placed in conjunction with its adhesive^12^. Clinical studies also compared a self-adhesive flowable composite to flowable composites applied with an adhesive. While the self-adhesive flowable composite was least retentive as a fissure sealant after 2 years (62.9%)^13^, there was no clinical difference between the flowable composites with or without self-adhesive formulation in minimally invasive Class I cavities of adult patients after 2 and 5 years, respectively^14,15^.

Flexural strength data, measured up to 1 year of water-storage in a three-point bending test according to ISO standard 4049, showed mean values above the acceptability threshold of 80 MPa defined in ISO 4049 for direct restorative materials^35^. In contrast to the manufacturer's long-term data and other in vitro studies showing comparable mechanical properties to clinically established posterior restoratives^19,21^, inferior outcomes were found by some investigators^36,37^. However, the 1-year recall results of the 35 class I and II self-adhesive bulk-fill restorations suggested that the material performs clinically acceptable in load-bearing areas of permanent teeth. The resulting failure rate was 2% after 1 year and 1.9% when calculated after the mean service time of 394 days. One clinical failure was reported in a three-surface class II restoration that the examining dentist believed was affected by occlusal factors, because both the restoration and the second upper molar partially fractured, and part of the restoration remained still bonded in place. Another two-surface class II restoration in the first upper molar in the same patient was found to be performing satisfactorily. After 1 year of clinical service, catastrophic failure rates for posterior restorations have been observed with some restorative materials. A composite releasing calcium, fluoride and hydroxyl ions showed a 6% failure rate^38^, a calcium aluminate cement a 16.7% failure rate^39^, and the use of the self-adhesive restorative Activa Bioactive-Restorative led to a 24.1% failure rate^16^, which indicates to follow-up new material categories also at shorter terms. In the present study, no clinical evidence for such catastrophic early failures of the restorations was found, supporting the good long-term mechanical stability of Surefil one class II restorations in vitro^19^. There is previous evidence that another self-adhesive resin-based bulk-fill restorative being not commercially available performed as well as a bulk-fill composite plus adhesive of the same manufacturer in class II cavities after 1 year^40^.

Color match was an aspect of the evaluation in which 12% unacceptable ratings (scores 3 and 4) were recorded after 1 year. The only shade A3 supplied at the prelaunch time of restoration placement was the principal reason for the color mismatch. The null hypothesis had to be rejected, because there were significant changes in color match over time. While 22 restorations were rated worse or showed no difference, 27 restorations were rated at least one score better at 1 year compared to baseline. Overall, 92% of the patients were satisfied with the appearance of their restorations. At the market launch of the self-adhesive bulk-fill restorative, five different shades were introduced. It can be assumed that the color mismatch that have been identified in the study would be improved by the wider shade range available in the commercially available product.

## Conclusion

The self-adhesive resin-based bulk-fill restorative Surefil one placed in a general dental practice setting showed acceptable short-term clinical results out to 1 year in stress-bearing class I and II as well as non-retentive class V cavities. The prospective clinical study will be continued to monitor these early findings.

## Data Availability

Upon reasonable request and according to ethical approval.
